# Towards individualized diagnostics of biofilm-associated infections: a case study

**DOI:** 10.1038/s41522-017-0030-5

**Published:** 2017-09-28

**Authors:** Mathias Müsken, Kathi Klimmek, Annette Sauer-Heilborn, Monique Donnert, Ludwig Sedlacek, Sebastian Suerbaum, Susanne Häussler

**Affiliations:** 1 Institute for Molecular Bacteriology, TWINCORE, Centre for Experimental and Clinical Infection Research, Hannover, Germany; 2 Department of Molecular Bacteriology, Helmholtz Centre for Infection Research, Braunschweig, Germany; 3 Department of Pulmonary Medicine, Hannover Medical School, Hannover, Germany; 4 Institute for Medical Microbiology and Hospital Epidemiology, Hannover Medical School, Hannover, Germany; 5 Present Address: Central Facility for Microscopy, Helmholtz Centre for Infection Research, Braunschweig, Germany; 6 Max von Pettenkofer Institute, Medical Microbiology and Hospital Epidemiology, München, Germany

## Abstract

Organized within biofilm communities, bacteria exhibit resistance towards a broad spectrum of antibiotics. Thus, one might argue that bacteria isolated from biofilm-associated chronic infections should be subjected to resistance profiling under biofilm growth conditions. Various test systems have been developed to determine the biofilm-associated resistance; however, it is not clear to what extent the in vitro results reflect the situation in vivo, and whether the biofilm-resistance profile should guide clinicians in their treatment choice. To address this issue, we used confocal microscopy in combination with live/dead staining, and profiled biofilm-associated resistance of a large number (>130) of clinical *Pseudomonas aeruginosa* isolates from overall 15 cystic fibrosis patients. Our results demonstrate that in addition to a general non-responsiveness of bacteria when grown under biofilm conditions, there is an isolate-specific and antibiotic-specific biofilm-resistance profile. This individual resistance profile is independent on the structural properties of the biofilms. Furthermore, biofilm resistance is not linked to the resistance profile under planktonic growth conditions, or a mucoid, or small colony morphology of the tested isolates. Instead, it seems that individual biofilm structures evolve during biofilm-associated growth and are shaped by environment-specific cues. In conclusion, our results demonstrate that biofilm resistance profiles are isolate specific and cannot be deduced from commonly studied phenotypes. Further clinical studies will have to show the added value of biofilm-resistance profiling. Individualized diagnosis of biofilm resistance might lead to more rational recommendations for antimicrobial therapy and, thus, increased effectiveness of the treatment of chronically infected patients.

## Biofilm resistance: Moving toward individualized testing

A procedure for individualized testing of biofilms might yield more reliable data on antibiotic susceptibility in clinical conditions. Researchers in Germany, led by Mathias Müsken at the Helmholtz Centre for Infection Research, developed a method to analyze the antibiotic resistance of patients’ biofilm samples, using microscopy and cell staining. They studied *Pseudomonas aeruginosa* biofilms gathered from people with cystic fibrosis. The procedure revealed differences in antibiotic susceptibility between bacteria growing in biofilms and in the free-living state. The findings cast doubt on the general utility of antibiotic resistance profiles derived from standard laboratory studies. The clinical advantages of using the procedure to study the antibiotic susceptibility of patients’ unique biofilms should be investigated further. The researchers suggest individualized testing might increase the effectiveness of treatment for chronically infected patients.

## Introduction

*Pseudomonas aeruginosa* plays an important role in pulmonary infections of cystic fibrosis (CF) patients.^1^ Despite intensified antimicrobial therapy, chronic *P. aeruginosa* lung infection, repeated exacerbations, and progressive deterioration in lung function remain a major cause of morbidity and mortality. In the chronically infected CF lung *P. aeruginosa* adopts a biofilm mode of growth that provides a protected niche for the bacteria.^2,3^ Biofilm bacteria are much more resistant to antibiotic treatment, as well as to the host immune response, and it has been shown that with the formation of bacterial biofilms it becomes difficult, if not impossible, to eradicate the infection.^4–8^

Antimicrobial susceptibility testing is applied to guide clinicians in their treatment choices. However, antibiotic susceptibilities of planktonic populations as determined by conventional susceptibility test methods may not reflect the actual resistance profile of biofilm-associated infections.^9^ The use of minimal inhibitory concentration (MIC) test results for the treatment of clinical exacerbations that are thought to be caused mainly by planktonic bacteria is likely valid. However, it might be valuable to complement the MIC results with a test aimed to decide, which is the best antibiotic to be used as a maintenance therapy in order to suppress the chronic infection.^3^

Various methods have been developed to determine antibiotic resistance under biofilm growth conditions.^10–13^ There have also been several attempts to evaluate their predictive values as diagnostic tools in clinical trials.^14–18^ However, it still remains to be shown that susceptibility testing under biofilm growth conditions results in different recommendations for antimicrobial treatment as compared to MIC testing and that chronically infected CF patients indeed benefit from biofilm resistance profiling.^19^

One of the major needs for the evaluation of the value of biofilm resistance profiling for clinical outcome is the use of a standardized and reliable high-throughput system to monitor biofilm growth under the addition of various antibiotics. We have previously developed an optical method to provide information on the responsiveness of *P. aeruginosa* biofilms to increasing concentrations of various antimicrobial agents.^20^ BacLight viability staining in combination with automated confocal laser scanning microscopy (CLSM) on *P. aeruginosa* grown under biofilm conditions in a 96-well plate format proved to be a highly effective and rapid method to monitor the efficiency of various antibiotics. Application of the optical system also revealed information on the structure and constitution of the bacterial biofilm population.

In this study, we optimized the optical method for antibiotic susceptibility profiling of biofilm-grown *P. aeruginosa* and determined biofilm-resistance profiles, as well as standard MIC and minimal bactericidal concentration (MBC) values of a large collection of clinical CF isolates. Most of the adult CF patients receive antibiotics for permanent inhalation therapy to suppress bacterial growth. Thus, we tested three antibiotics which are currently available for inhalation: aztreonam, colistin, and tobramycin. High concentrations of these antibiotics can be reached within the CF lung despite significant inter-patient variation.^21–26^

Our results demonstrate that there are isolate-specific and antibiotic-specific biofilm-resistance profiles and that the individual profiles cannot be deduced from other isolate-specific characteristics, such as the structure of the biofilm or the MIC values of the individual clinical isolates. An individualized diagnostics of biofilm-associated resistance might therefore overcome the limitations of conventional resistance testing for the prediction of treatment success/failure and thus might improve quality of health care measures in chronically infected patients.

## Results

### Collection of clinical isolates and determination of the biofilm-active score (BAS)

Overall, 113* P. aeruginosa* isolates were collected from 15 CF patients over a period of two and a half years (Table S1). An additional 20 isolates were sampled as a follow up for 13 out of 15 patients. On average, nine *P. aeruginosa* isolates were obtained from each patient with a minimum of five isolates and a maximum of 15. Some of the patient´s sputum samples contained a morphologically diverse population including small colony variants (SCVs) or mucoid isolates, others contained only one morphotype. The recovery of a morphological diverse population from chronically infected sites is well described and seems to reflect bacterial adaptation to hostile environments.^27–29^

**Fig. 1:**
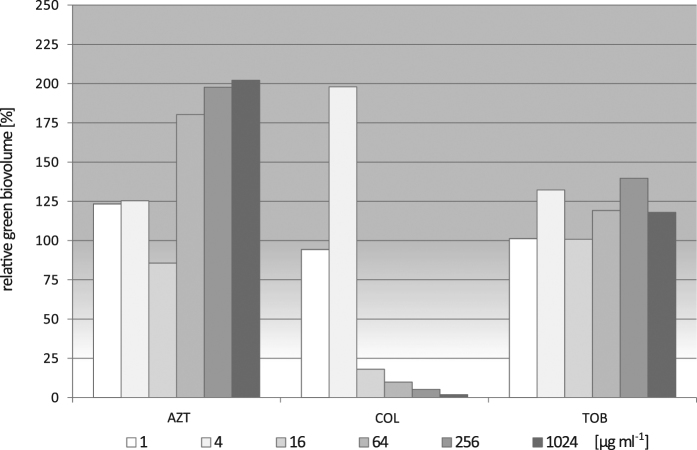
Determination of the biofilm-active score (BAS) of the three antibiotics on *P. aeruginosa* biofilm-grown bacteria. An exemplary data set on the responsiveness of biofilm-grown bacteria of the clinical isolate Iso1525 towards various concentrations of the antibiotics aztreonam (AZT), colistin (COL), and tobramycin (TOB) is shown. We used the untreated control to set the corresponding green biovolume values as the 100% vitality value. The green biovolume can increase to values larger than 100%, if biofilm formation is induced by non-lethal antibiotic concentrations (a common observation). The isolate Iso1525 is non-responsive towards the activity of AZT and TOB, but concentrations ≥16 µg ml^−1^ COL lead to a reduction in the green biovolume by >75%. This corresponds to a BAS of +++. The BAS is dependent on the green biovolume reduction and the minimal antibiotic concentration necessary to reach the reduction. For details of BAS categorizations see Table S2

We grew the 133 clinical isolates within biofilms in 96-well plates and subjected them to serial dilutions of the inhalative antibiotics aztreonam, colistin and tobramycin. Following a live and dead staining, those biofilms were analyzed using CLSM. The green (live) biovolume was monitored and the relative reduction of the green biovolume upon antibiotic treatment was determined. As exemplified in Fig. 1, we categorized the biofilm as “fully responsive” (with a BAS of +++) if the green biovolume was reduced by >75% within a certain antibiotic concentration range (please see Table S2 for details), as “responsive” if the biovolume was reduced in the range of 50–75% (BAS++), as “weakly responsive” in the range 25–50% (BAS+), and as “non-responsive”/resistant (R) if a reduction of the green biovolume of less than 25% was observed. As demonstrated previously,^20^ biofilm-resistance profiling was very robust and a concentration-dependent reduction could be observed for both, the green biovolume and the colony forming unit (CFU) counts (Fig. S1).

### Comparison of antibiotic effectiveness on planktonic and biofilm-grown bacteria

**Fig. 2:**
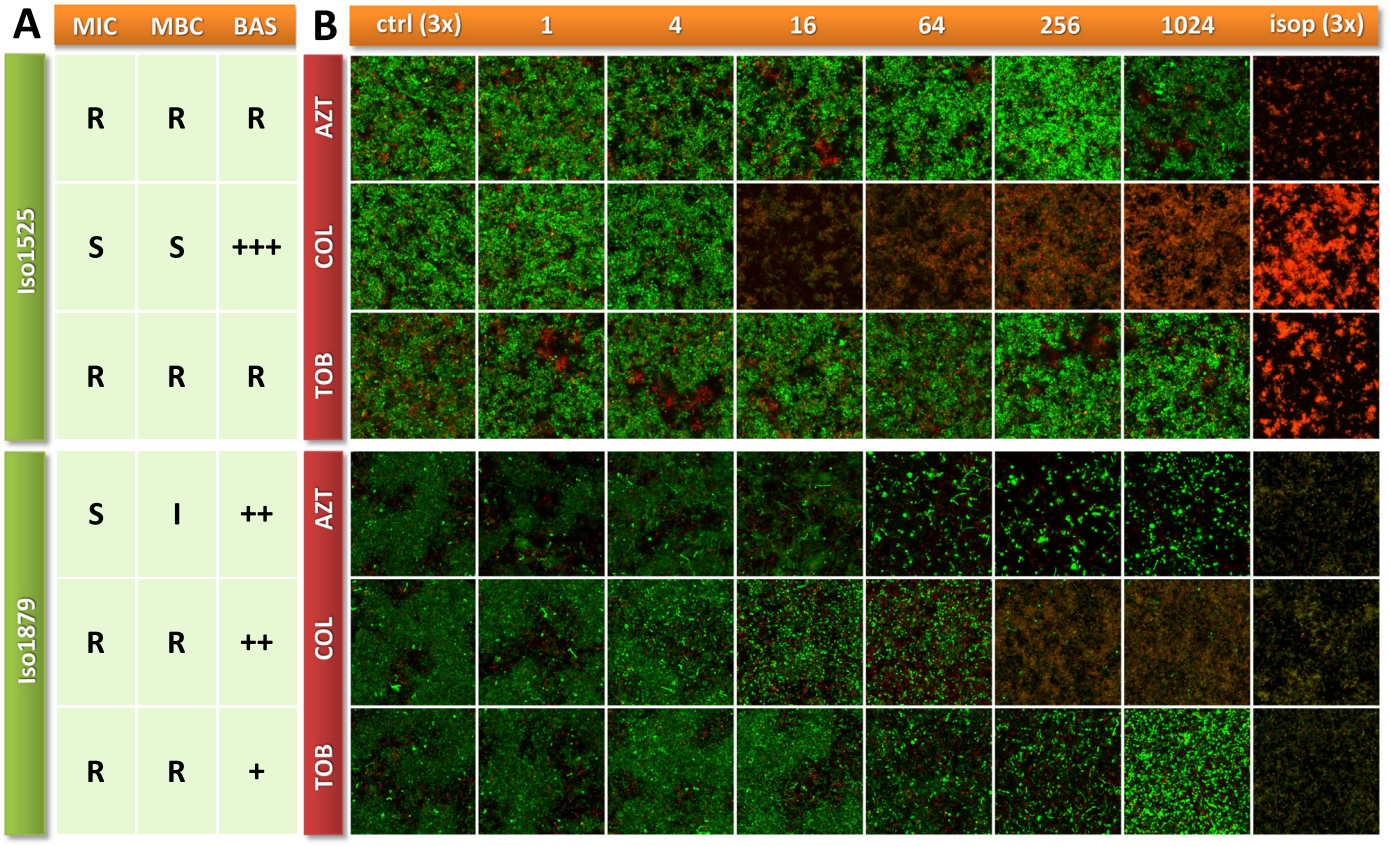
Comparison of antibiotic resistance profiles. **a** Minimal inhibitory/bactericidal concentrations (MIC/MBC) and the biofilm-active score (BAS) of aztreonam (AZT), colistin (COL), and tobramycin (TOB) of the two clinical isolates Iso1525 and Iso0052. **b** Biofilm projections of single samples treated with increasing concentrations of antibiotic [µg ml^−1^]. ctrl: non-treated control, isop: isopropanol control (triplicates). Forty-eight hours old biofilms are stained with the BacLight^TM^ Viability-Kit, visualizing dead cells in red (propidium iodide) and living cells in green (Syto9)

Examples of acquired biofilm images and resistance data of two clinical isolates (Iso1525 and Iso0052) are presented in Fig. 2. The isolate Iso1525 exhibited MIC/MBC values in the resistant range for aztreonam and tobramycin, whereas the MIC values for colistin were in the sensitive range. As might have been expected, the biofilm-grown bacteria of the isolate were non-responsive towards the activity of aztreonam and tobramycin, but responsive towards the activity of colistin at the given concentration range.

Isolate Iso0052 exhibited MIC values in the resistant range towards the activity of colistin and tobramycin, and values in the sensitive range towards the activity of aztreonam. However, all the three antibiotics were effective as we observed an overall reduction in the green biofilm biovolume.

In Table S3, a summary of MIC, MBC, and BAS values for the 133 clinical isolates of all 15 patients is shown. Additionally, colony morphology characteristics (SCV, mucoidity) are recorded. Many of the isolates were very sensitive and showed MIC and MBC values below the tested antibiotic concentration range. 1, 51, and 4 of the clinical isolates exhibited MIC and MBC values of ≤1 µg ml^−1^ towards aztreonam, colistin, and tobramycin, respectively. Other isolates were highly resistant and exhibited MIC values above the tested concentration range of ≥128 µg ml^−1^ for aztreonam (43), colistin (5) and tobramycin (16 of the clinical isolates). For those isolates MBC values were not determined.

In general, the antibiotic concentration required to effectively kill the planktonically growing bacteria (MBC) was usually higher than the MIC (2 to ≥8 times higher). Only in three clinical isolates the MIC and MBC were identical. A strongly increased MBC as compared to the MIC (MBC/MIC: ≥8×) was mainly found for aztreonam and colistin in a number of strains (42 of 89 (47%) and 33 of 76 (43%), respectively), this number was two times lower for tobramycin (24 of 113 (21%) isolates).

Most of the aztreonam resistant (MIC) clinical isolates were also non-responsive towards the antimicrobial activity if grown under biofilm conditions (50 out of 62 isolates, Table 1). In addition, 21 out of 23 aztreonam intermediate resistant isolates were resistant towards the activity of aztreonam if grown under biofilm conditions. These results corborate previous findings of an increased resistance of isolates grown under biofilm conditions. However, 12 of the resistant (according to their MIC) and two of the intermediate resistant isolates were responsive towards aztreonam under biofilm growth conditions. Vice versa, from the overall 22 aztreonam-sensitive isolates (MIC values of ≤1 µg ml^−1^), 14 isolates (Table 1) were resistant towards the activity of aztreonam if grown under biofilm conditions and eight were responsive.

**Table 1:** Comparison of antibiotic resistance profiles

	MIC		MBC		BAS		Percentage (%)
Aztreonam (107 isolates included)	Resistant	62	Resistant	62	Resistant	50	80.6
			Intermediate	0	+	7	11.3
			Sensitive	0	++	2	3.2
					+++	3	4.8
	Intermediate	23	Resistant	21	Resistant	21	91.3
			Intermediate	2	+	1	4.3
			Sensitive	0	++	1	4.3
					+++		0.0
	Sensitive	22	Resistant	0	Resistant	14	63.6
			Intermediate	22^a^	+	3	13.6
			Sensitive	0	++	5	22.7
					+++	0	0.0
Tobramycin (114 isolates incl.)	Resistant	40	Resistant	40	Resistant	28	70.0
					+	6	15.0
			Sensitive	0	++	5	12.5
					+++	1	2.5
	Sensitive	74	Resistant	39	Resistant	49	66.2
					+	8	10.8
			Sensitive	35	++	10	13.5
					+++	7	9.5
Colistin (115 isolates incl.)	Resistant	13	Resistant	13	Resistant	1	7.7
					+	0	0.0
			Sensitive	0	++	3	23.1
					+++	9	69.2
	Sensitive	102	Resistant	23	Resistant	1	1.0
					+	7	6.9
			Sensitive	79	++	33	32.4
					+++	61	59.8

^a^ The MBC of 16 isolates was ≥8 µg ml^−1^ and, thus, either intermediate or resistant

Similar results were obtained for tobramycin (Table 1). Twenty-eight of the 40 clinical strains that exhibited MIC values in the resistant range were non-responsive towards tobramycin under biofilm-growth conditions, whereas 12 strains proved to be responsive (BAS of +, ++, or +++). Vice versa 49 of the 74 tobramycin-sensitive isolates were non-responsive towards the activity of tobramycin if cultured under biofilm conditions, whereas 25 were responsive.

Colistin seems to effectively kill the bacteria even under biofilm-growth conditions (Table 1). 92% of the colistin resistant isolates (12 of 13 isolates) exhibited a BAS of at least ++. On the other hand, only 1 of the 102 colistin-sensitive isolates exhibited a BAS in the non-responsive range. More than 53% (61 out of 115) of the clinical isolates were fully responsive (BAS+++) and more than 29% (33 out of 115) were responsive (BAS++) towards the activity of colistin (total: 82%) if grown under biofilm conditions. In contrast only 10% (11 out of 107) and 20% (23 out of 114) of the clinical isolates exhibited a BAS of ++ or +++ towards the activity of aztreonam and tobramycin, respectively.

Our results thus demonstrate that resistance values increase if the bacteria are grown under biofilm conditions. However, some clinical strains exhibit an unexpected responsiveness or non-responsiveness towards antibiotic activity under biofilm growth conditions.

### Link between colony morphologies and planktonic and biofilm-resistance patterns

To evaluate whether the colony morphologies impact on resistance profiles, we compared the planktonic and biofilm-resistance profiles of the mucoid (*n* = 25 isolates from overall eight patients) and SCV (*n* = 38, 12 patients) isolates to that of the whole community (*n* = 100, 33 isolates have been excluded due to missing BAS values).

**Fig. 3:**
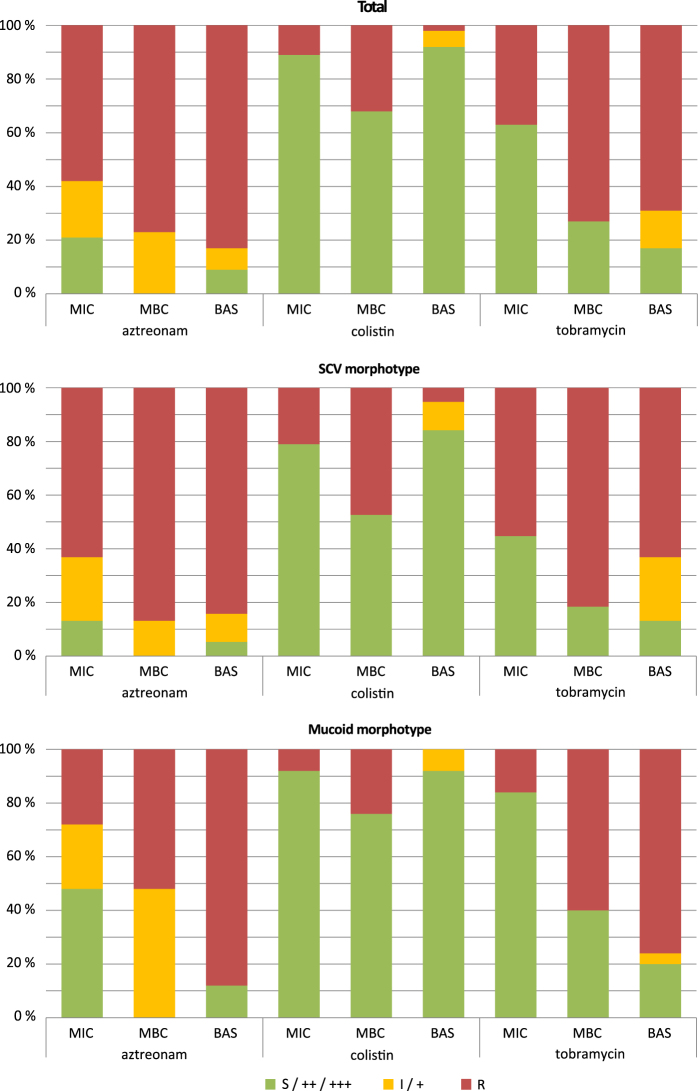
Planktonic but not biofilm-resistance profiles differed between morphotypes. The distribution of susceptible (S), intermediate (I), and resistant (R) isolates towards the respective antibiotic are shown. The BAS categories ++ and +++ were defined as susceptible and category + as intermediate. In **a** data of all isolates (*n* = 100) is shown, in **b** and **c** the data of SCVs (*n* = 38) and the mucoid morphotype (*n* = 25) are shown, respectively. Both morphotypes did not overlap since there was no mucoid SCV

As depicted in Fig. 3, the SCV phenotype was associated with higher MIC values, whereas the mucoid strains were more susceptible as compared to the overall population against all three of the tested antibiotics. This higher susceptibility of mucoid isolates has been described before.^30,31^ However, in contrast to the planktonic conditions, mucoid isolates seem to be as resistant or even slightly more antibiotic resistant as compared to the overall population if grown under biofilm conditions.

### Correlation between the *P. aeruginosa* biofilm structure, colony morphology, and the biofilm-resistance profile

**Fig. 4:**
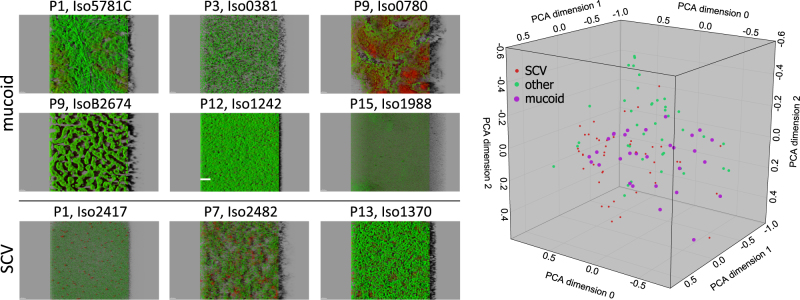
Mucoid or SCV clinical isolates do not form specific biofilm structures. **a** Examples of biofilm phenotypes of selected mucoid and SCV morphotypes visualized in an easy-3D projection (48 h-old biofilms stained with the BacLight™ Viability-Kit) and **b** principal component analysis (PCA) representation of the biofilm phenotype of 113 clinical isolates. Seven biofilm parameters were taken into account. SCV are depicted in red, mucoid in pink, and others in green

**Fig. 5:**
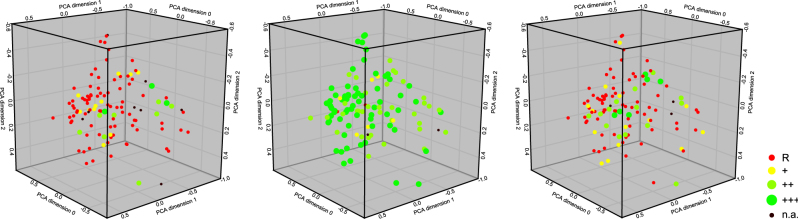
Expression of distinct biofilm structures do not correlate with particular biofilm-active scores. PCA representation of the biofilm phenotype of 113 clinical isolates. The BAS categories for **a** aztreonam, **b** colistin, and **c** tobramycin are depicted. The colors represent resistant (red) isolates and isolates with a BAS category of + (yellow), ++ (light green), and +++ (green)

Within the collection of 133 isolates from the overall 15 CF patients, not only different colony morphotypes were observed, but also morphologically distinct biofilm phenotypes as determined by CLSM (Fig. S2). Some of the biofilms were rather flat, whereas others exhibited distinctive structures. Of note, there did not seem to be a correlation between colony morphology (SCV, mucoid and other) and the biofilm phenotype. The various mucoid or SCV isolates did not exhibit specific biofilm structures compared to the other clinical isolates (Fig. 4). Furthermore, we did not observe a correlation of the biofilm structure and the biofilm-active scores (Fig. 5).

### *P. aeruginosa* biofilm structure across different CF patients over time

In general, as expected, biofilm phenotypes of the infecting *P. aeruginosa* isolates exhibited various biofilm phenotypes across the different CF patients (high inter-patient variability). Furthermore, in some patients the various isolates that were recovered from one sputum sample exhibited quite distinct biofilm phenotypes (intra-patient variability) (Figs. S2 and S3). Nevertheless similar biofilm phenotypes also appeared recurrently in different patients, indicating that there are biofilm phenotypes (as they become apparent in the in vitro system) that are formed again and again (Fig. S4).

Interestingly, in most of the patients the biofilm phenotypes were stable and could be repeatedly observed in subsequently isolated *P. aeruginosa* strains (e.g. patients 11, 12, and 14, Fig. S2). Furthermore, isolates from the same patients tended to form similar biofilm structures even if they expressed different colony morphotypes (patients 7 and 12, Fig. S3). This indicates that the patient-specific microenvironment seems to shape the biofilm phenotype and that this microenvironment can be found repeatedly in the lungs of various CF patients.

## Discussion

We have recently established a method, which allows susceptibility testing of biofilm-grown *P. aeruginosa* isolates. In this study, the method was adjusted to susceptibility testing of the inhaled antimicrobial agents aztreonam, colistin, and tobramycin. Susceptibility profiles of 133 biofilm-grown clinical *P. aeruginosa* isolates, recovered from the respiratory tract material of overall 15 CF patients were determined. We aimed to address the question of whether an individual testing of the resistance profile under biofilm-growth conditions might be justified. Biofilm-resistance profiling might be worth the effort, if biofilm-resistance profiles cannot be inferred from the MIC values.

A plethora of studies have previously demonstrated that biofilm growth protects the bacteria from the activity of a many different antimicrobials.^5,32–37^ In agreement with this, we observed that the inhalative antibiotics were generally less effective under biofilm-growth conditions. However, we also observed strain-dependent differences in the effectiveness of the inhalative antibiotics under biofilm-growth conditions and those differences were independent of the MIC profiles. The discordance of MIC and BAS profiles was most apparent for aztreonam and tobramycin. 19.4% of the aztreonam and 30.0% of the tobramycin resistant (according to the MIC) isolates were still responsive to the antimicrobials under biofilm-growth conditions, whereas 63.6 and 66.2% of the sensitive (according to the MIC) isolates were non-responsive under biofilm-growth conditions. Of note, whereas many clinical isolates were almost completely non-responsive towards the activity of aztreonam and tobramycin under biofilm-growth conditions, colistin seemed to be fully active. The bactericidal activity of colistin against *P. aeruginosa* biofilms has been shown to be enhanced under anaerobic/microaerophilic conditions,^38^ which are also found in microtiter plate assays,^39,40^ as well as in CF lungs.^41^

Our results clearly demonstrate that in order to increase antibiotic effectiveness in chronic biofilm-associated infections, only individual testing of the biofilm-resistance profile will give the full information on which antibiotic could be most effective. This holds particularly true for the inhalative antibiotics, aztreonam and tobramycin, and seems to be less relevant for colistin.

Interestingly, testing of the biofilm responsiveness towards the activity of the three antibiotics revealed no clear cross-resistance. This indicates that mechanisms of biofilm resistance are, in addition to a general tolerance of biofilm-grown bacteria towards external stresses at least in parts due to strain-specific characteristics, to develop resistance against single antibiotics. In this study, we also observed that the colony morphologies impacted the MIC profiles. Mucoid isolates were generally more sensitive and SCVs more resistant to either of the antibiotics tested in this study. This phenomenon has been described before.^30,42–46^ In contrast, colony morphologies did not impact the biofilm structure, nor did they influence the responsiveness of biofilm-grown bacteria towards the activity of the inhalative antibiotics.

This underscores our finding that biofilm resistance is indeed independent on the MIC profile and that the individual infecting *P. aeruginosa* isolates do exhibit distinct biofilm responsiveness that is intrinsically and stably linked to the bacterial genotype.

In conclusion, in this pilot study we have carefully evaluated biofilm susceptibility profiles of a collection of clinical isolates. Our finding of clearly discordant results between planktonic and biofilm resistances implies that individual testing of the resistance profiles under biofilm-growth conditions might be worth the effort in those clinical isolates, which have been recovered from a chronic biofilm-associated infected site. Biofilm-resistance profiling as a novel diagnostic measure might significantly impact on a more rational and more effective antimicrobial inhalative therapy of chronically *P. aeruginosa* infected CF patients. Nevertheless, large clinical trials will be required in order to demonstrate that biofilm-resistance profiles indeed lead to a more reliable positive predictive value for clinical success/failure of inhaled antimicrobial therapy.

## Methods

### Bacterial cultivation

Clinical *P. aeruginosa* isolates were isolated from respiratory tract samples at the Institute of Medical Microbiology and Hospital Epidemiology at the Hannover Medical School and collected over a period of 2.5 years (2010–2012) from 15 patients. In total, 113 isolates could be recovered. Additional 20 isolates from 13 patients (no additional isolates from patients 6 and 7) have been collected ~3 years after the initial study period. Isolates were routinely cultivated on Columbia agar plates with 5% sheep blood (Becton Dickinson) to determine the colony morphotype (mucoid and SCV). Glycerol stocks were stored at −80 °C until use.

### Determination of the MIC and MBC

For MIC determinations, single colonies of overnight grown isolates were suspended in 0.9% NaCl. Optical densities (OD_600_) were adjusted to 0.01 and mixed with serially diluted antibiotics in LB medium within a microtiter plate to a final cell concentration in the well of 5 × 10^6^ cells ml^−1^. The antibiotic test ranges of aztreonam, colistin, and tobramycin were 1, 2, 4, 8, 16, 32, and 64 µg ml^−1^. After overnight cultivation (~16 h), the microtiter plates were visually inspected and the MIC (the lowest antibiotic concentration that prevented visible growth) was recorded. Clinical breakpoints were defined according to the European Committee on Antimicrobial Susceptibility Testing (EUCAST) classification to categorize susceptible (S) and resistant (R) isolates, including intermediate resistant (I) isolates for aztreonam.

For MBC determinations, we spotted 100 µl (total volume) of the MIC-plate wells without visible growth (MIC and two higher concentrations) into single wells of a 24-well plates filled with LB agar (1.5%). Growth was evaluated after a minimum of 24 h of incubation (plates were kept longer to allow evaluation of slow growing isolates, e.g. SCVs). The lowest antibiotic concentration without visible growth was recorded as the MBC. All experiments were performed in triplicates.

### Determination of biofilm resistance

Biofilm susceptibility testing was performed as previously described.^20^ Briefly, bacterial pre-cultures were grown in LB and diluted to an OD_600_ of 0.002. Biofilm formation in LB medium accommodated growth of the majority of clinical isolates and proved to be stable and robust. Hundred microliters per well was used as inoculum for biofilm growth in a µclear half area plate (Greiner Bio-one). Bacteria were allowed to establish a biofilm for 24 h at 37 °C in a humid atmosphere. Bacterial biofilms were then exposed to antibiotics at the following concentrations: 1, 4, 16, 64, 256, and 1024 µg ml^−1^. The addition of distilled water and isopropanol served as a growth and killing control, respectively. In parallel, dyes of the BacLight Viability kit (Molecular Probes, Inc.) were added to differentiate live (Syto9) and dead (propidium iodide) bacteria. After another growth period of 24 h, bacteria were analyzed via confocal laser-scanning microscopy (CLSM). In one 96-well plate, resistance profiles of two isolates were tested against three antibiotics (Fig. S5).

To determine CFUs, dilution series (1:10) of resuspended biofilms were spotted onto agar plates using a 96-pin replicator.

### Confocal microscopy

Automated microscopy was performed with an inverted SP8 system (Leica Microsystems) and the Leica application suite LAS X including the Matrix screener tool. To obtain biofilm images, two image stacks were acquired in parallel at the center of a well. Both stacks were acquired with a 40×/NA 1.1 water objective and the same laser and detector settings. However, stack 1 (overview job) was acquired using a zoom × 0.75 (image size: ~387 × 387 µm, pixel size of 0.378 µm) and a total range of ~80 µm (27 slides and a slice distance of 3 µm), while stack 2 (zoom job) was acquired with a zoom × 4 (image size: ~73 µm × 73 µm; pixel size of 0.142 µm) to visualize single cells. The stack size of the zoom job was reduced to 30 µm (10 slides × 3 µm). Both stacks started at position zero which is the substratum (foil) found by a reflection-based autofocus run. For the automated image acquisition of all samples, a number of pre-defined laser/detector settings were assigned to compensate inter-species and inter-well fluctuations in intensity avoiding under-exposed and over-exposed images.

### Image-analysis and visualization

Both acquired image stacks were analyzed with the Developer XD (Definiens) software. The programmed customized solution of the Developer software is based on a previously described software called PHLIP^47^ and is used to determine biofilm-specific parameter for both fluorescent channels separately, as well as combined including, for example, ratios of the differentially stained populations within the biofilm or the biovolume, which describes the biofilm biomass. To optimize the analysis, we combined data of the overview and the zoom image stacks; the biovolume was taken from the overview job to define the overall amount of biofilm, while we used the percentage of dead (red fluorescent) cells of the zoom job. The latter has a better image resolution and, thus, allowed a more accurate estimation of fluorescence ratios. With both values we determined the green fluorescent biofilm—the living population—and compared treated samples with the control. A series of antibiotic dilutions are needed to categorize the responsiveness. ImageJ and Imaris (Bitplane AG) were used for the visualization of biofilm image stacks, the latter for complex 3D reconstructions.

### Principal component analysis (PCA)

Seven biofilm parameters (biovolume [µm^3^], area to volume [µm^−1^], horizontal and vertical spreading [µm^2^], mean thickness [µm], roughness [–], and substrate coverage bottom [%]) of a combined fluorescence channel (including fluorescence of green and red signals) were used for PCA using the software KNIME (version 3.3.1^48^). In addition to the untreated controls (three samples), samples treated with 1 µg ml^−1^ antibiotic (three samples) have been included to determine more robust mean values of the biofilm parameters for each isolate, since the biofilm phenotype was not influenced by low antibiotic concentration (see Figs. 1 and 2). In total, 20 isolates have been excluded from PCA analysis due to missing image data in half of the replicas (weak green fluorescence in controls; contaminants in region of interest) or erroneous data analysis (BAS determination failed in minimum two of three antibiotics).

### Data availability

The data that support the findings of this study are available from the corresponding author upon reasonable request.

## Electronic supplementary material

Supplementary Information

Suppl. Figure S2
