# Supplementary material for: Towards individualized diagnostics of biofilm-associated infections: a case study
Source: NPJ Biofilms Microbiomes. 2017 Sep 28;3:22. doi: 10.1038/s41522-017-0030-5 (PMC5620081; doi:10.1038/s41522-017-0030-5)
Supplement: Supplementary file 1 — Supplementary Information [file 41522_2017_30_MOESM1_ESM.docx]

# Supplementary Information for:

Towards individualized diagnostics of biofilm-associated infections: A case study

**Mathias Müsken^*^, Kathi Klimmek, Annette Sauer-Heilborn, Monique Donnert, Ludwig Sedlacek, Sebastian Suerbaum, Susanne Häussler**

*corresponding author: mathias.muesken@helmholtz-hzi.de

Helmholtz Centre for Infection Research, Department of Molecular Bacteriology, Inhoffenstrasse 7, 38124 Braunschweig, Germany

## Supplementary tables

Table S1: Clinical isolates collected over a period of 2.5 years (9 (Q)uarters)

|  | 2010 | | | 2011 | | | | 2012 | | 2015 | morphotypes | | | isolates |
| --- | --- | --- | --- | --- | --- | --- | --- | --- | --- | --- | --- | --- | --- | --- |
| **Patient** | **Q1** | **Q2** | **Q3** | **Q4** | **Q5** | **Q6** | **Q7** | **Q8** | **Q9** |  | **SCV** | **mucoid** | **others** | total |
| **1** |  | 1 |  |  | 1 | 1 |  | 2 |  | 3 | 7 | 0 | 1 | 8 |
| **2** |  |  |  | 1 |  | 1 |  | 1 |  | 2 | 1 | 1 | 3 | 5 |
| **3** |  | 2 | 1 | 2 |  |  | 2 |  |  | 1 | 0 | 7 | 1 | 8 |
| **4** |  |  |  |  |  | 2 | 1 | 1 |  | 3 | 0 | 0 | 7 | 7 |
| **5** |  |  | 1 | 2 |  |  | 1 |  |  | 1 | 2 | 2 | 1 | 5 |
| **6** |  |  |  | 3 |  |  | 2 | 2 |  |  | 2 | 2 | 3 | 7 |
| **7** | 2 | 1 |  | 1 | 1 |  |  | 5 | 3 |  | 7 | 2 | 4 | 13 |
| **8** | 1 |  | 1 |  |  | 1 | 1 | 2 | 2 | 1 | 2 | 1 | 6 | 9 |
| **9** | 2 | 1 | 1 |  | 1 | 1 | 1 | 2 | 1 | 1 | 1 | 10 | 0 | 11 |
| **10** |  | 2 | 1 |  | 2 |  |  | 2 | 2 | 2 | 7 | 0 | 4 | 11 |
| **11** |  |  |  |  | 1 | 1 | 1 | 1 |  | 1 | 0 | 5 | 0 | 5 |
| **12** |  | 2 | 1 | 1 | 2 | 2 | 1 | 2 | 3 | 1 | 5 | 0 | 10 | 15 |
| **13** | 1 |  |  |  | 2 | 1 | 2 |  | 2 | 1 | 6 | 1 | 2 | 9 |
| **14** |  | 1 | 1 | 3 |  | 2 |  | 2 |  | 2 | 2 | 0 | 9 | 11 |
| **15** |  |  |  |  | 1 | 2 | 2 | 2 | 1 | 1 | 1 | 4 | 4 | 9 |
|  |  |  |  |  |  |  |  |  |  |  |  |  |  | 133 |

Table S2: Definition of the four categories of the biofilm-active score: BAS

| **r(esistant)** | | **cat. +** | | **cat. ++** | | **cat. +++** | |
| --- | --- | --- | --- | --- | --- | --- | --- |
| BR* | AB-conc. [µg/ml] | BR* | AB-conc. [µg/ml] | BR* | AB-conc. [µg/ml] | BR* | AB-conc. [µg/ml] |
| <25% | all |  |  |  |  |  |  |
| 25-50% | 256, 1024 | 25-50% | 16, 64 | 25-50% | 1, 4 |  |  |
|  |  | 50-75% | 256, 1024 | 50-75% | 16, 64 | 50-75% | 1, 4 |
|  |  |  |  | >75% | 256, 1024 | >75% | 16, 64 |

cat.: category; BR*: green biovolume reduction

| **Table S3: Summary of plate morphotypes and antibiotic resistance profiles of all tested clinical isolates** | **Morphotypes** | | **Aztreonam** | | | | **Colistin** | | | | **Tobramycin** | | | |
| --- | --- | --- | --- | --- | --- | --- | --- | --- | --- | --- | --- | --- | --- | --- |
| **Patient _isolate** | **SCV** | **mucoid** | **MIC** | **MBC** | **MBC/MIC** | **BAS** | **MIC** | **MBC** | **MBC/MIC** | **BAS** | **MIC** | **MBC** | **MBC/MIC** | **BAS** |
| **P01_Iso0473** | x |  | 16 | 32 | 2x | R | ≤ 1 | 4 | ≥ 4x | +++ | ≤ 1 | ≥ 8 | ≥ 8x | R |
| **P01_Iso1414** | x |  | 64 | ≥ 128 | ≥ 2x | R | ≤ 1 | 4 | ≥ 4x | +++ | 16 | 32 | 2x | R |
| **P01_Iso1676** |  |  | ≤ 1 | ≤ 1 | n.d. | n.a. | ≤ 1 | ≤ 1 | n.d. | n.a. | ≤ 1 | ≤ 1 | n.d. | n.a. |
| **P01_Iso2417** | x |  | 32 | ≥ 128 | ≥ 4x | R | 4 | ≥ 32 | ≥ 8x | +++ | 16 | 32 | 2x | R |

| **P01_Iso2418** | x |  | 32 | ≥ 128 | ≥ 4x | R | 2 | ≥ 16 | ≥ 8x | +++ | 16 | 32 | 2x | R |
| --- | --- | --- | --- | --- | --- | --- | --- | --- | --- | --- | --- | --- | --- | --- |
| **P01_Iso5321#** | x |  | ≥ 128 | n.d. | n.d. | R | 8 | 32 | 4x | +++ | 32 | 32 | 1x | + |
| **P01_IsoB2724#** | x |  | 2 | 16 | 8x | R | ≤ 1 | ≤ 1 | n.d. | +++ | ≤ 1 | 2 | ≥ 2x | R |

| **P01_IsoB2725#** | x |  | 32 | ≥ 128 | ≥ 4x | R | ≤ 1 | 8 | ≥ 8x | +++ | 32 | 64 | 2x | R |
| --- | --- | --- | --- | --- | --- | --- | --- | --- | --- | --- | --- | --- | --- | --- |
| **P02_Iso0985** |  |  | ≥ 128 | n.d. | n.d. | R | ≤ 1 | ≤ 1 | n.d. | ++ | 16 | 64 | 4x | R |
| **P02_Iso1669** |  |  | ≥ 128 | n.d. | n.d. | R | ≤ 1 | ≤ 1 | n.d. | +++ | 16 | 32 | 2x | R |
| **P02_Iso2203** |  |  | 16 | 64 | 4x | R | ≤ 1 | ≤ 1 | n.d. | ++ | 2 | 4 | 2x | R |
| **P02_Iso5781B#** | x |  | ≥ 128 | n.d. | n.d. | + | ≥ 128 | n.d. | n.d. | R | ≥ 128 | n.d. | n.d. | R |
| **P02_Iso5781C#** |  | x | ≥ 128 | n.d. | n.d. | R | ≤ 1 | 2 | ≥ 2x | ++ | 2 | 8 | 4x | ++ |
| **P03_Iso0381** |  | x | ≤ 1 | ≥ 8 | ≥ 8x | R | ≤ 1 | ≤ 1 | n.d. | +++ | ≤ 1 | ≤ 1 | n.d. | R |
| **P03_Iso0382** |  | x | ≤ 1 | 4 | ≥ 4x | R | ≤ 1 | ≥ 8 | ≥ 8x | +++ | ≤ 1 | ≤ 1 | n.d. | R |
| **P03_Iso0668** |  | x | ≤ 1 | ≥ 8 | ≥ 8x | n.a. | ≤ 1 | 4 | ≥ 4x | n.a. | ≤ 1 | ≥ 8 | ≥ 8x | n.a. |
| **P03_Iso0928** |  | x | ≤ 1 | ≥ 8 | ≥ 8x | ++ | 64 | ≥ 128 | ≥ 2x | +++ | ≤ 1 | 4 | ≥ 4x | +++ |
| **P03_Iso0929** |  |  | ≤ 1 | ≥ 8 | ≥ 8x | ++ | ≤ 1 | ≤ 1 | n.d. | +++ | ≤ 1 | ≥ 8 | ≥ 8x | +++ |
| **P03_Iso1883** |  | x | ≤ 1 | ≥ 8 | ≥ 8x | n.a. | ≤ 1 | ≤ 1 | n.d. | n.a. | ≤ 1 | 2 | ≥ 2x | n.a. |
| **P03_Iso1884** |  | x | ≤ 1 | ≥ 8 | ≥ 8x | ++ | ≤ 1 | ≤ 1 | n.d. | +++ | ≤ 1 | ≤ 1 | n.d. | R |
| **P03_IsoB2726#** |  | x | ≥ 128 | n.d. | n.d. | R | ≤ 1 | ≤ 1 | n.d. | ++ | 4 | 8 | 2x | R |
| **P04_Iso1721** |  |  | 32 | 64 | 2x | n.a. | ≤ 1 | ≤ 1 | n.d. | n.a. | 2 | ≥ 16 | ≥ 8x | n.a. |
| **P04_Iso1722** |  |  | 16 | ≥ 128 | ≥ 8x | n.a. | ≤ 1 | ≥ 8 | ≥ 8x | n.a. | 16 | ≥ 128 | ≥ 8x | n.a. |
| **P04_Iso2066** |  |  | 32 | ≥ 128 | ≥ 4x | R | ≤ 1 | ≥ 8 | ≥ 8x | +++ | 2 | 8 | 4x | R |
| **P04_Iso2419** |  |  | 2 | 8 | 4x | R | ≤ 1 | ≤ 1 | n.d. | +++ | 4 | ≥ 32 | ≥ 8x | R |
| **P04_IsoB2616#** |  |  | 32 | ≥ 128 | ≥ 4x | R | ≤ 1 | ≥ 8 | ≥ 8x | ++ | 2 | 8 | 4x | R |
| **P04_IsoB2617#** |  |  | ≤ 1 | ≥ 8 | ≥ 8x | R | ≤ 1 | 4 | ≥ 4x | ++ | ≤ 1 | ≥ 8 | ≥ 8x | R |
| **P04_IsoB2618#** |  |  | 64 | ≥ 128 | ≥ 2x | R | ≤ 1 | ≥ 8 | ≥ 8x | ++ | 16 | 64 | 4x | R |
| **P05_Iso0703** |  | x | 4 | ≥ 32 | ≥ 8x | R | ≤ 1 | ≤ 1 | n.d. | +++ | ≤ 1 | ≥ 8 | ≥ 8x | R |
| **P05_Iso1076** | x |  | ≥ 128 | n.d. | n.d. | + | ≤ 1 | ≤ 1 | n.d. | n.a. | ≤ 1 | 2 | ≥ 2x | ++ |
| **P05_Iso1093** |  |  | ≥ 128 | n.d. | n.d. | n.a. | 32 | ≥ 128 | ≥ 4x | n.a. | ≤ 1 | 4 | ≥ 4x | n.a. |
| **P05_Iso2100** | x |  | ≥ 128 | n.d. | n.d. | + | ≤ 1 | ≤ 1 | n.d. | +++ | ≤ 1 | ≥ 8 | ≥ 8x | ++ |
| **P05_Iso5759#** |  | x | ≤ 1 | ≥ 8 | ≥ 8x | R | ≤ 1 | ≤ 1 | n.d. | ++ | ≤ 1 | 8 | ≥ 8x | R |
| **P06_Iso0925** | x |  | ≥ 128 | n.d. | n.d. | n.a. | 2 | 4 | 2x | +++ | ≥ 128 | n.d. | n.d. | ++ |
| **P06_Iso0926** |  |  | ≥ 128 | n.d. | n.d. | R | 2 | 4 | 2x | +++ | ≥ 128 | n.d. | n.d. | R |
| **P06_Iso0927** |  |  | ≥ 128 | n.d. | n.d. | R | 2 | 2 | 1x | +++ | 2 | 4 | 2x | + |
|  | **Morphotypes** | | **Aztreonam** | | | | **Colistin** | | | | **Tobramycin** | | | |
| **Patient _isolate** | **SCV** | **mucoid** | **MIC** | **MBC** | **MBC/MIC** | **BAS** | **MIC** | **MBC** | **MBC/MIC** | **BAS** | **MIC** | **MBC** | **MBC/MIC** | **BAS** |
| **P06_Iso1858** |  |  | 4 | 8 | 2x | R | ≤ 1 | ≤ 1 | n.d. | +++ | 4 | 8 | 2x | R |
| **P06_Iso1859** |  | x | 2 | 8 | 4x | n.a. | ≤ 1 | ≤ 1 | n.d. | +++ | ≤ 1 | 2 | ≥ 2x | n.a. |
| **P06_Iso2472** |  | x | 2 | 8 | 4x | n.a. | ≤ 1 | ≤ 1 | n.d. | +++ | 2 | 4 | 2x | n.a. |
| **P06_Iso2474** | x |  | ≥ 128 | n.d. | n.d. | R | ≤ 1 | 4 | ≥ 4x | ++ | ≥ 128 | n.d. | n.d. | R |
| **P07_Iso0147** |  | x | 64 | ≥ 128 | ≥ 2x | R | ≤ 1 | 4 | ≥ 4x | +++ | 4 | 16 | 4x | ++ |
| **P07_Iso0149** | x |  | 32 | ≥ 128 | ≥ 4x | R | ≤ 1 | 4 | ≥ 4x | +++ | ≤ 1 | ≥ 8 | ≥ 8x | R |
| **P07_Iso0440** | x |  | 32 | ≥ 128 | ≥ 4x | R | ≤ 1 | 2 | ≥ 2x | ++ | 4 | 8 | 2x | + |
| **P07_Iso1149** |  |  | 16 | ≥ 128 | ≥ 8x | R | ≤ 1 | 2 | ≥ 2x | ++ | 4 | 8 | 2x | + |
| **P07_Iso1495** |  |  | 16 | ≥ 128 | ≥ 8x | n.a. | 2 | 8 | ≥ 4x | ++ | 4 | 16 | 4x | R |
| **P07_Iso2220** | x |  | ≥ 128 | n.d. | n.d. | R | ≤ 1 | ≤ 1 | n.d. | + | ≤ 1 | 2 | ≥ 2x | + |
| **P07_Iso2221** |  |  | 32 | ≥ 128 | ≥ 4x | R | ≤ 1 | ≤ 1 | n.d. | n.a. | 2 | 8 | 4x | R |
| **P07_Iso2479*** | x |  | 32 | ≥ 128 | ≥ 4x | R | ≤ 1 | 2 | ≥ 2x | ++ | 2 | ≥ 16 | ≥ 8x | R |
| **P07_Iso2482** | x |  | 64 | ≥ 128 | ≥ 2x | R | 2 | 4 | 2x | ++ | ≤ 1 | ≥ 8 | ≥ 8x | ++ |
| **P07_Iso2483** |  |  | 32 | ≥ 128 | ≥ 4x | n.a. | ≤ 1 | ≤ 1 | n.d. | + | 4 | 16 | 4x | n.a. |
| **P07_Iso2744** |  | x | ≥ 128 | n.d. | n.d. | ++ | ≤ 1 | 4 | ≥ 4x | ++ | 4 | 16 | 4x | + |
| **P07_Iso2748** | x |  | 64 | ≥ 128 | ≥ 2x | R | ≤ 1 | ≤ 1 | n.d. | + | ≤ 1 | 4 | ≥ 4x | R |
| **P07_Iso2749** | x |  | ≥ 128 | n.d. | n.d. | +++ | 4 | ≥ 32 | ≥ 8x | ++ | 8 | 32 | 4x | +++ |
| **P08_Iso0004** |  |  | 64 | ≥ 128 | ≥ 2x | n.a. | ≤ 1 | 2 | ≥ 2x | n.a. | ≤ 1 | 2 | ≥ 2x | +++ |
| **P08_Iso0721** |  |  | 64 | ≥ 128 | ≥ 2x | n.a. | ≤ 1 | 4 | ≥ 4x | ++ | ≤ 1 | 2 | ≥ 2x | ++ |
| **P08_Iso1525** |  |  | ≥ 128 | n.d. | n.d. | R | ≤ 1 | ≤ 1 | n.d. | +++ | ≥ 128 | n.d. | n.d. | R |
| **P08_Iso1879** |  | x | 16 | ≥ 64 | ≥ 4x | R | ≤ 1 | ≥ 8 | ≥ 8x | n.a. | ≤ 1 | ≥ 8 | ≥ 8x | +++ |
| **P08_Iso2336** |  |  | ≥ 128 | n.d. | n.d. | R | ≤ 1 | ≤ 1 | n.d. | ++ | ≥ 128 | n.d. | n.d. | R |
| **P08_Iso2337** |  |  | 64 | ≥ 128 | ≥ 2x | R | ≤ 1 | ≤ 1 | n.d. | +++ | ≥ 128 | n.d. | n.d. | R |
| **P08_Iso2610** | x |  | 64 | ≥ 128 | ≥ 2x | R | ≤ 1 | ≥ 8 | ≥ 8x | +++ | ≥ 128 | n.d. | n.d. | R |
| **P08_Iso2611** |  |  | 16 | ≥ 128 | ≥ 8x | n.a. | ≤ 1 | 2 | ≥ 2x | n.a. | 2 | 4 | 2x | n.a. |
| **P08_IsoB2733#** | x |  | 16 | ≥ 128 | ≥ 8x | R | ≤ 1 | 8 | ≥ 8x | + | ≥ 128 | n.d. | n.d. | R |
| **P09_Iso0070** |  | x | ≤ 1 | ≥ 8 | ≥ 8x | R | ≤ 1 | ≤ 1 | n.d. | +++ | ≤ 1 | 2 | ≥ 2x | R |
| **P09_Iso0071** |  | x | ≤ 1 | 4 | ≥ 4x | R | ≤ 1 | ≤ 1 | n.d. | ++ | ≤ 1 | 2 | ≥ 2x | R |
| **P09_Iso0459** |  | x | ≤ 1 | ≥ 8 | ≥ 8x | n.a. | ≤ 1 | ≤ 1 | n.d. | ++ | ≤ 1 | 2 | ≥ 2x | R |
| **P09_Iso0780** |  | x | 8 | ≥ 64 | ≥ 8x | R | ≤ 1 | 2 | ≥ 2x | ++ | 2 | 8 | 4x | R |
| **P09_Iso1435** |  | x | 8 | ≥ 64 | ≥ 8x | ++ | ≤ 1 | ≤ 1 | n.d. | +++ | ≤ 1 | 2 | ≥ 2x | n.a. |
| **P09_Iso1769** |  | x | 8 | ≥ 64 | ≥ 8x | R | ≤ 1 | ≥ 8 | ≥ 8x | ++ | ≤ 1 | 4 | ≥ 4x | R |
| **P09_Iso2125** |  | x | 8 | ≥ 64 | ≥ 8x | n.a. | ≤ 1 | 2 | ≥ 2x | +++ | ≤ 1 | 4 | ≥ 4x | R |
| **P09_Iso2411** |  | x | 4 | ≥ 32 | ≥ 8x | R | ≤ 1 | ≤ 1 | n.d. | + | ≤ 1 | 4 | ≥ 4x | R |
| **P09_Iso2412** |  | x | 32 | 64 | 2x | ++ | ≤ 1 | ≤ 1 | n.d. | ++ | ≤ 1 | 2 | ≥ 2x | n.a. |

| **P09_Iso2822** | x |  | 4 | ≥ 32 | ≥ 8x | R | ≤ 1 | ≤ 1 | n.d. | R | ≤ 1 | 2 | ≥ 2x | R |
| --- | --- | --- | --- | --- | --- | --- | --- | --- | --- | --- | --- | --- | --- | --- |
|  | **Morphotypes** | | **Aztreonam** | | | | **Colistin** | | | | **Tobramycin** | | | |
| **Patient _isolate** | **SCV** | **mucoid** | **MIC** | **MBC** | **MBC/MIC** | **BAS** | **MIC** | **MBC** | **MBC/MIC** | **BAS** | **MIC** | **MBC** | **MBC/MIC** | **BAS** |
| **P09_IsoB2674#** |  | x | 16 | ≥ 128 | ≥ 8x | R | ≤ 1 | ≤ 1 | n.d. | ++ | ≤ 1 | 4 | ≥ 4x | R |
| **P10_Iso0212** |  |  | ≤ 1 | ≥ 8 | ≥ 8x | + | 2 | ≥ 16 | ≥ 8x | +++ | 8 | 16 | 2x | + |
| **P10_Iso0213** | x |  | ≤ 1 | ≥ 8 | ≥ 8x | R | ≥ 128 | n.d. | n.d. | +++ | 16 | 64 | 4x | R |
| **P10_Iso0696** | x |  | ≤ 1 | ≥ 8 | ≥ 8x | n.a. | 8 | ≥ 64 | ≥ 8x | +++ | 64 | ≥ 128 | ≥ 2x | R |
| **P10_Iso1512** |  |  | ≥ 128 | n.d. | n.d. | + | ≤ 1 | ≥ 8 | ≥ 8x | +++ | 32 | 64 | 2x | R |
| **P10_Iso1513** | x |  | ≥ 128 | n.d. | n.d. | R | 8 | ≥ 64 | ≥ 8x | +++ | 64 | ≥ 128 | ≥ 2x | ++ |
| **P10_Iso2249** | x |  | ≥ 128 | n.d. | n.d. | n.a. | 16 | 64 | ≥ 4x | +++ | 64 | ≥ 128 | ≥ 2x | R |
| **P10_Iso2256** |  |  | ≤ 1 | ≥ 8 | ≥ 8x | ++ | ≤ 1 | ≥ 8 | ≥ 8x | ++ | 8 | 16 | 2x | ++ |
| **P10_Iso2573** |  |  | ≥ 128 | n.d. | n.d. | R | ≤ 1 | 4 | ≥ 4x | +++ | 4 | 16 | 4x | R |
| **P10_Iso2574** | x |  | ≥ 128 | n.d. | n.d. | + | 2 | ≥ 16 | ≥ 8x | +++ | 64 | ≥ 128 | ≥ 2x | n.a. |
| **P10_Iso5510_4#** | x |  | ≥ 128 | n.d. | n.d. | R | 2 | ≥ 16 | ≥ 8x | +++ | 16 | 64 | 4x | + |
| **P10_Iso5510_5#** | x |  | ≥ 128 | n.d. | n.d. | R | 2 | ≥ 16 | ≥ 8x | +++ | 32 | 64 | 2x | + |
| **P11_Iso1380** |  | x | ≤ 1 | ≥ 8 | ≥ 8x | R | ≤ 1 | ≥ 8 | ≥ 8x | +++ | ≤ 1 | ≥ 8 | ≥ 8x | R |
| **P11_Iso1692** |  | x | ≤ 1 | ≥ 8 | ≥ 8x | R | ≤ 1 | 2 | ≥ 2x | +++ | 2 | 8 | 4x | R |
| **P11_Iso2059** |  | x | ≤ 1 | ≥ 8 | ≥ 8x | R | ≤ 1 | ≥ 8 | ≥ 8x | +++ | 2 | ≥ 16 | ≥ 8x | R |
| **P11_Iso2332** |  | x | ≤ 1 | ≥ 8 | ≥ 8x | + | ≤ 1 | ≤ 1 | n.d. | n.a. | ≤ 1 | 2 | ≥ 2x | R |
| **P11_Iso5431_3#** |  | x | ≤ 1 | ≥ 8 | ≥ 8x | R | 8 | 8 | 1x | ++ | 8 | 8 | 1x | R |
| **P12_Iso0426** |  |  | ≥ 128 | n.d. | n.d. | R | ≤ 1 | ≤ 1 | n.d. | +++ | 2 | 4 | 2x | R |
| **P12_Iso0427** |  |  | 64 | ≥ 128 | ≥ 2x | R | ≥ 128 | n.d. | n.d. | ++ | 32 | 64 | 2x | R |
| **P12_Iso0731** |  |  | ≥ 128 | n.d. | n.d. | R | ≤ 1 | ≤ 1 | n.d. | +++ | ≤ 1 | 4 | ≥ 4x | R |
| **P12_Iso1037** |  |  | ≥ 128 | n.d. | n.d. | R | ≤ 1 | ≤ 1 | n.d. | +++ | 16 | 32 | 2x | R |
| **P12_Iso1242** |  |  | ≥ 128 | n.d. | n.d. | R | ≤ 1 | ≤ 1 | n.d. | +++ | 4 | 8 | 2x | R |
| **P12_Iso1244** |  |  | ≥ 128 | n.d. | n.d. | n.a. | ≤ 1 | ≤ 1 | n.d. | +++ | 2 | 2 | 1x | R |
| **P12_Iso1664** |  |  | ≥ 128 | n.d. | n.d. | R | ≤ 1 | ≤ 1 | n.d. | +++ | 4 | 8 | 2x | R |
| **P12_Iso1665** |  |  | ≥ 128 | n.d. | n.d. | R | ≤ 1 | ≤ 1 | n.d. | +++ | ≤ 1 | ≥ 8 | ≥ 8x | + |
| **P12_Iso2001** |  |  | ≥ 128 | n.d. | n.d. | R | ≤ 1 | ≤ 1 | n.d. | +++ | ≤ 1 | 2 | ≥ 2x | R |
| **P12_Iso2344** | x |  | ≥ 128 | n.d. | n.d. | R | ≤ 1 | ≤ 1 | n.d. | +++ | 2 | 8 | 4x | R |
| **P12_Iso2345** |  |  | ≥ 128 | n.d. | n.d. | R | ≤ 1 | ≤ 1 | n.d. | +++ | ≤ 1 | ≥ 8 | ≥ 8x | R |
| **P12_Iso2684** | x |  | ≥ 128 | n.d. | n.d. | R | ≤ 1 | ≤ 1 | n.d. | +++ | ≤ 1 | 2 | ≥ 2x | R |
| **P12_Iso2685** | x |  | 4 | ≥ 32 | ≥ 8x | R | ≤ 1 | ≤ 1 | n.d. | +++ | ≤ 1 | 2 | ≥ 2x | + |
| **P12_Iso2688** | x |  | 4 | ≥ 32 | ≥ 8x | R | ≤ 1 | ≤ 1 | n.d. | +++ | ≤ 1 | ≥ 8 | ≥ 8x | R |
| **P12_Iso5724#** | x |  | ≥ 128 | n.d. | n.d. | R | ≤ 1 | ≤ 1 | n.d. | +++ | 4 | 8 | 2x | R |
| **P13_Iso0052** | x |  | ≤ 1 | 4 | ≥ 4x | ++ | ≥ 128 | n.d. | n.d. | ++ | ≥ 128 | n.d. | n.d. | + |
| **P13_Iso1370** | x |  | 32 | ≥ 128 | ≥ 4x | R | ≤ 1 | 1 | n.d. | +++ | 4 | 8 | 2x | R |
| **P13_Iso1371** | x |  | 16 | ≥ 128 | ≥ 8x | + | 16 | ≥ 128 | ≥ 8x | +++ | ≥ 128 | n.d. | n.d. | R |
| **P13_Iso1658** | x |  | 16 | ≥ 128 | ≥ 8x | R | 16 | ≥ 128 | ≥ 8x | +++ | ≥ 128 | n.d. | n.d. | + |
|  | **Morphotypes** | | **Aztreonam** | | | | **Colistin** | | | | **Tobramycin** | | | |
| **Patient _isolate** | **SCV** | **mucoid** | **MIC** | **MBC** | **MBC/MIC** | **BAS** | **MIC** | **MBC** | **MBC/MIC** | **BAS** | **MIC** | **MBC** | **MBC/MIC** | **BAS** |
| **P13_Iso2009** |  |  | 16 | ≥ 128 | ≥ 8x | R | ≤ 1 | 4 | ≥ 4x | +++ | 4 | ≥ 32 | ≥ 8x | ++ |
| **P13_Iso2010** | x |  | ≤ 1 | 4 | ≥ 4x | + | ≤ 1 | ≥ 8 | ≥ 8x | +++ | ≥ 128 | n.d. | n.d. | ++ |
| **P13_Iso2545** | x |  | 4 | ≥ 32 | ≥ 8x | R | 4 | ≥ 32 | ≥ 8x | +++ | ≥ 128 | n.d. | n.d. | R |
| **P13_Iso2548** |  |  | 16 | ≥ 128 | ≥ 8x | R | ≤ 1 | 4 | ≥ 4x | +++ | 1 | ≥ 8 | ≥ 8x | R |
| **P13_Iso5708#** |  | x | 16 | ≥ 128 | ≥ 8x | R | ≤ 1 | 2 | ≥ 2x | ++ | 2 | 4 | 2x | R |
| **P14_Iso0373** |  |  | 32 | ≥ 128 | ≥ 4x | +++ | ≤ 1 | 2 | ≥ 2x | +++ | 2 | 4 | 2x | R |
| **P14_Iso0711** |  |  | 8 | 16 | 2x | n.a. | ≤ 1 | ≤ 1 | n.d. | n.a. | 64 | ≥ 128 | ≥ 2x | n.a. |
| **P14_Iso1077** |  |  | 64 | ≥ 128 | ≥ 2x | R | ≤ 1 | 4 | ≥ 4x | ++ | 2 | 8 | 4x | R |
| **P14_Iso1078** |  |  | ≥ 128 | n.d. | n.d. | n.a. | ≤ 1 | ≥ 8 | ≥ 8x | n.a. | 2 | 4 | 2x | n.a. |
| **P14_Iso1079** |  |  | 32 | ≥ 128 | ≥ 4x | + | ≤ 1 | 2 | ≥ 2x | ++ | 2 | 4 | 2x | ++ |
| **P14_Iso1570** |  |  | ≥ 128 | n.d. | n.d. | + | ≤ 1 | ≥ 8 | ≥ 8x | +++ | 2 | 4 | 2x | +++ |
| **P14_Iso1572** |  |  | ≥ 128 | n.d. | n.d. | +++ | ≤ 1 | 4 | ≥ 4x | ++ | 2 | 4 | 2x | ++ |
| **P14_Iso2420** |  |  | 32 | ≥ 128 | ≥ 4x | n.a. | ≤ 1 | 4 | ≥ 4x | ++ | ≤ 1 | 4 | ≥ 4x | +++ |
| **P14_Iso2441** |  |  | ≥ 128 | n.d. | n.d. | R | ≤ 1 | 4 | ≥ 4x | +++ | 2 | 4 | 2x | +++ |
| **P14_Iso5864_1#** | x |  | ≤ 1 | 4 | ≥ 4x | R | ≤ 1 | ≤ 1 | n.d. | + | 64 | ≥ 128 | ≥ 2x | R |
| **P14_Iso5864_2#** | x |  | ≤ 1 | 8 | ≥ 8x | R | ≤ 1 | 2 | ≥ 2x | ++ | ≤ 1 | 2 | ≥ 2x | + |
| **P15_Iso1397** |  | x | 32 | ≥ 128 | ≥ 4x | R | ≤ 1 | 2 | ≥ 2x | + | 16 | ≥ 128 | ≥ 8x | R |
| **P15_Iso1822** |  |  | 32 | 64 | 2x | n.a. | 2 | ≥ 16 | ≥ 8x | n.a. | ≥ 128 | n.d. | n.d. | n.a. |
| **P15_Iso1823** |  |  | 64 | ≥ 128 | ≥ 2x | n.a. | 4 | ≥ 32 | ≥ 8x | n.a. | 64 | ≥ 128 | ≥ 2x | n.a. |
| **P15_Iso1985** |  |  | 64 | ≥ 128 | ≥ 2x | n.a. | 4 | ≥ 32 | ≥ 8x | n.a. | 64 | ≥ 128 | ≥ 2x | n.a. |
| **P15_Iso1988** |  | x | 64 | ≥ 128 | ≥ 2x | R | 2 | 4 | 2x | ++ | 32 | 64 | 2x | R |
| **P15_Iso2322** |  | x | ≥ 128 | n.d. | n.d. | R | ≤ 1 | 4 | ≥ 4x | ++ | 16 | ≥ 128 | ≥ 8x | ++ |
| **P15_Iso2323** |  | x | ≤ 1 | ≥ 8 | ≥ 8x | R | ≤ 1 | 2 | ≥ 2x | +++ | ≤ 1 | ≥ 8 | ≥ 8x | ++ |
| **P15_Iso2613** |  |  | 64 | ≥ 128 | ≥ 2x | n.a. | 4 | ≥ 32 | ≥ 8x | n.a. | 64 | ≥ 128 | ≥ 2x | n.a. |
| **P15_IsoB2734#** | x |  | ≥ 128 | n.d. | n.d. | R | ≥ 128 | n.d. | n.d. | +++ | ≥ 128 | n.d. | n.d. | R |
| # recent isolates; *: nose sample; n.d.: not detectable; n.a.: no / weak analysis (grey) | | | | | | |  |  |  |  |  |  |  |  |
| MIC / MBC: EUCAST category S (green), I (yellow) and R (red) | | | | | |  |  |  |  |  |  |  |  |  |
| BAS category: +++ (green), ++ (light green), + (yellow), R (red) | | | | | |  |  |  |  |  |  |  |  |  |
|  |  |  |  |  |  |  |  |  |  |  |  |  |  |  |

## Supplementary figures

**Figure S1**


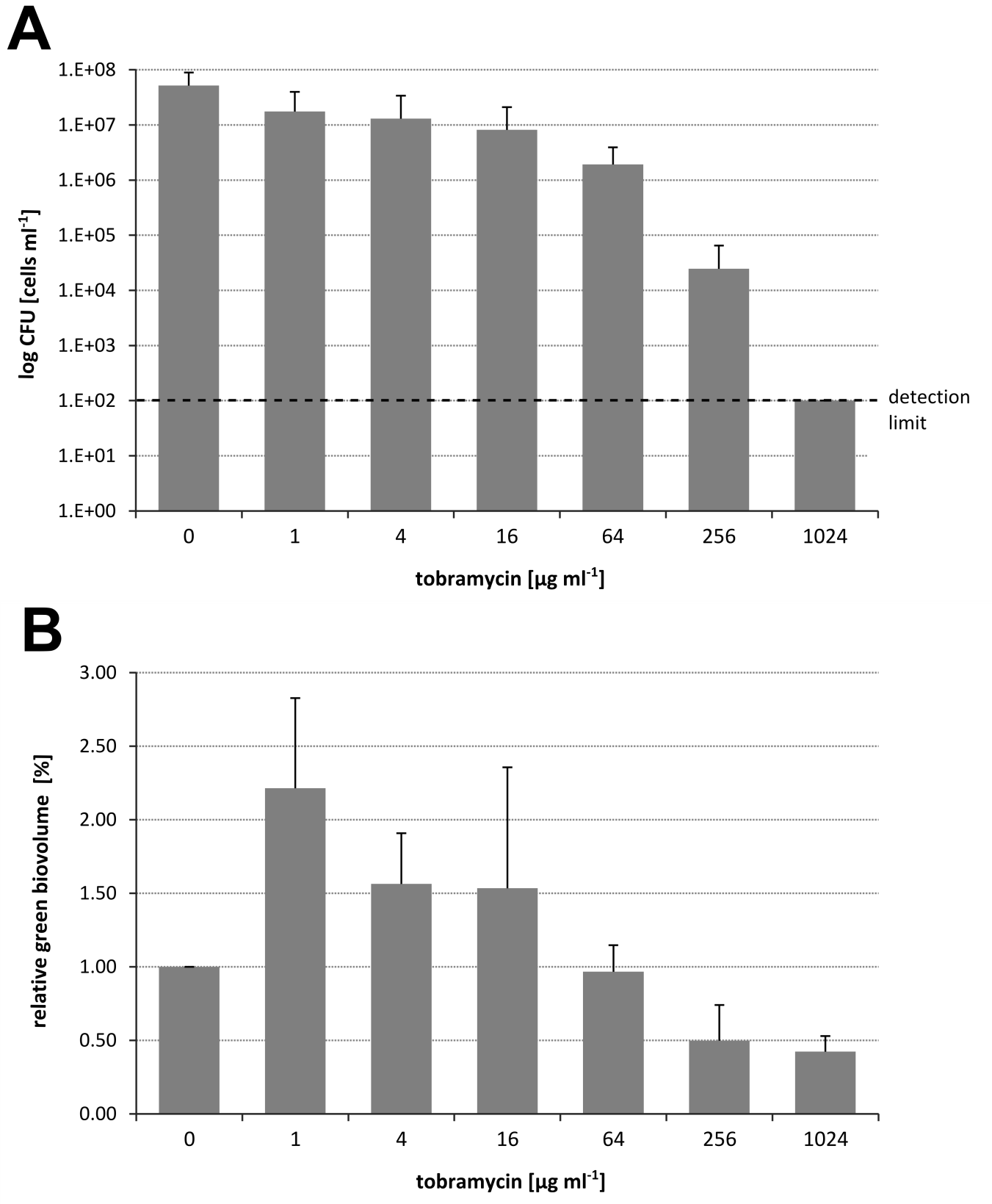


**Figure S1: Reproducibility of the anti-biofilm activity in the *in vitro* test system.** PA14 biofilms were subjected to various concentrations of tobramycin and CFU counts (A) and image analysis (B) were performed as measures of the overall responsiveness of the bacterial population to the antibiotic in the given concentration range. In (A) data are the mean of 6 biological (each with 3 technical) replica. In (B), the relative green biovolume values of 6 biological (each with 2 technical) replica are depicted.

Despite the fact that both assays (live/dead staining and CFU determination) show overall similar concentration-dependent responsiveness of the bacterial population to the antibiotic, the values may diverge for individual antibiotic concentrations. While propidium iodide (red) staining of cells indicates loss of membrane integrity (direct measurement in the presence of antibiotic stress), CFUs determinations represent the number of bacteria capable to recover if antibiotic stress is withdrawn.

**Figure S2**

**(s. extra file)**

**Figure S2: Representative Easy-3D projections of untreated biofilms of all isolates in the time frame of this project (quarter 1-9 and later)**. The 48 h old biofilms are stained with the BacLight™ Viability-Kit, visualizing dead cells in red (propidium iodide) and living cells in green (Syto9). Colony morphotype information as well as planktonic and biofilm resistance profiles are indicated (see example highlighted in red).

**Figure S3**


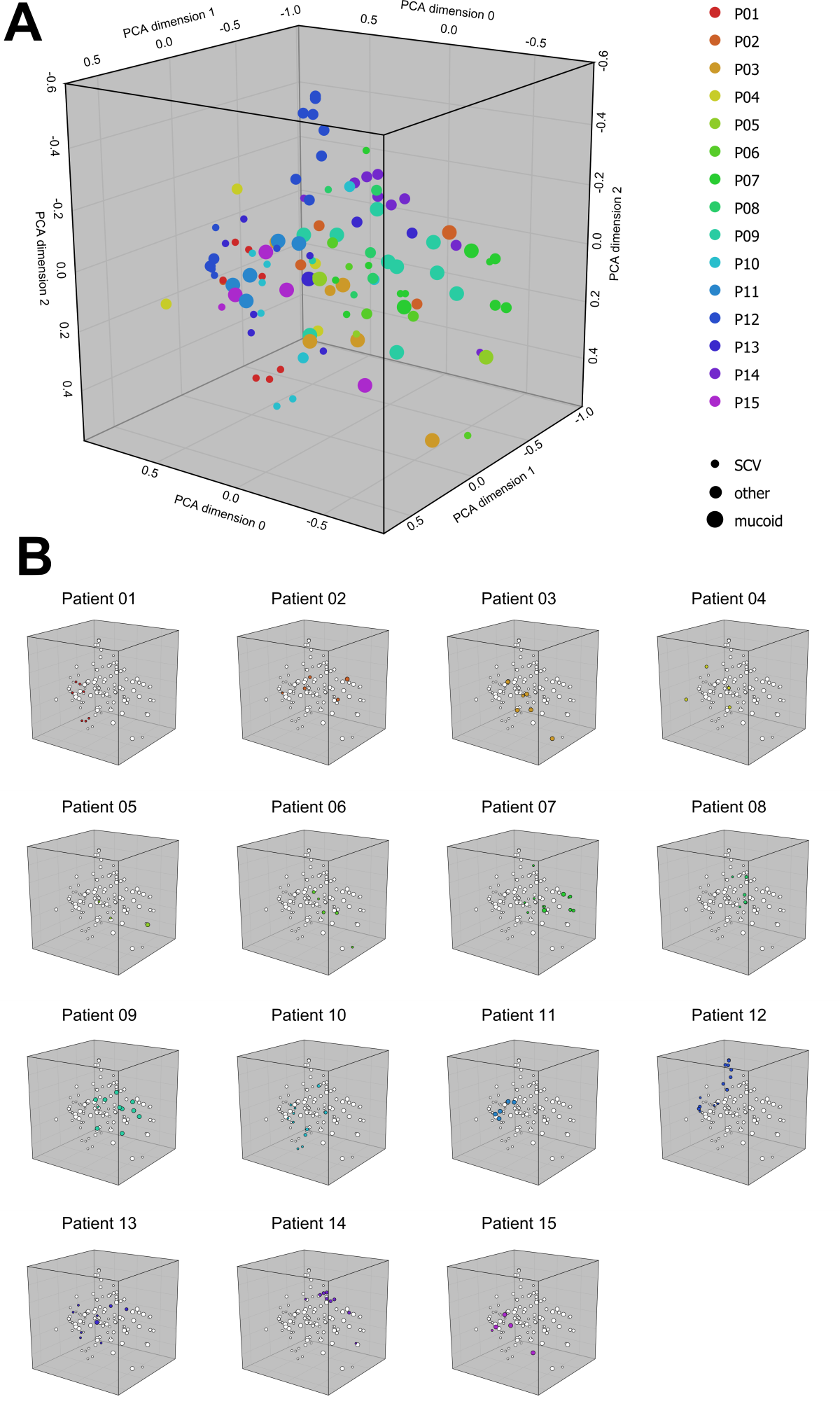


**Figure S3:** **Biofilm phenotype distribution is patient dependent.** Biofilm phenotypes were described by the following 7 parameter: biovolume, area to volume, horizontal and vertical spreading, mean thickness, roughness, and substrate coverage at the bottom. Principal component analysis (PCA) representation of the biofilm phenotype of 113 clinical isolates is shown. Isolates recovered from one patient are indicated by identical color in a graph depicting all patients (A) or separated (B) from each other. The different colony morphotypes vary in size: SCV (small), other (medium) and mucoid (large).

**Figure S4**


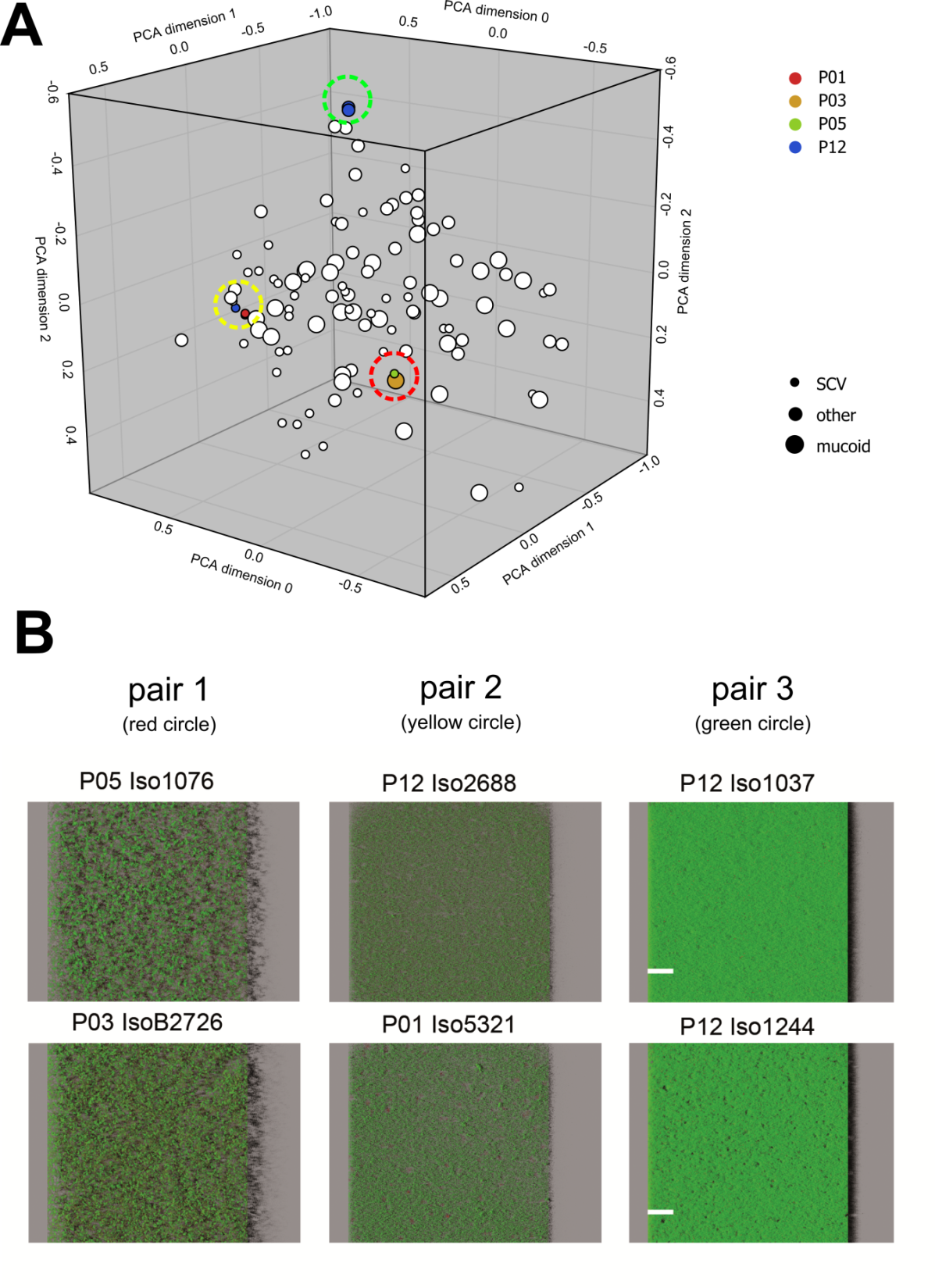


**Figure S4:** **Examples of isolates with similar biofilm phenotypes.** (A) PCA of the biofilm phenotype as shown in Figure S3 highlighting three pairs of clinical isolates. Colors represent patient numbers and the different cycle sizes the various colony morphotypes. (B) Easy-3D projections of the selected biofilm phenotype pairs. The 48 h old biofilms are stained with the BacLight™ Viability-Kit, visualizing dead cells in red (propidium iodide) and living cells in green (Syto9).

**Figure S5**

**Figure S5: 96-well plate layout for the biofilm susceptibility testing.** Antibiotics aztreonam (AZT), colistin (COL) and tobramycin (TOB) are tested in serial dilutions. In addition, triplicates of the water control (ctrl) and isopropanol control (isop) are tested.
