# Supplementary material for: Towards individualized diagnostics of biofilm-associated infections: a case study
Source: NPJ Biofilms Microbiomes. 2017 Sep 28;3:22. doi: 10.1038/s41522-017-0030-5 (PMC5620081; doi:10.1038/s41522-017-0030-5)
Supplement: Supplementary file 2 — Suppl. Figure S2 [file 41522_2017_30_MOESM2_ESM.pdf]

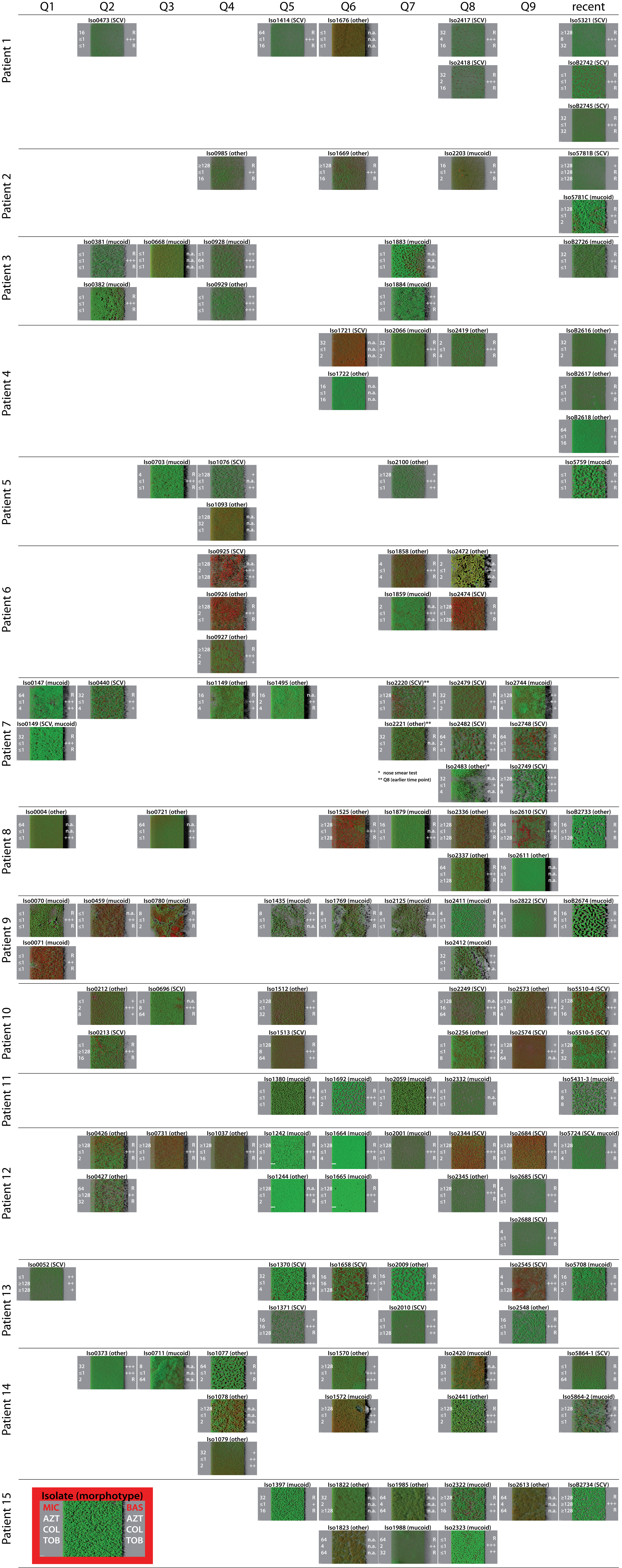

Q1
Q2
Q3
Q4
Q5
Q6
Q7
Q8
Q9
recent
Patient 1
Iso0473 (SCV)
Iso1414 (SCV)
Iso1676 (other)
Iso2417 (SCV)
Iso2418 (SCV)
Iso5321 (SCV)
IsoB2742 (SCV)
IsoB2745 (SCV)
Patient 2
Iso0985 (other)
Iso1669 (other)
Iso2203 (mucoid)
Iso5781B (SCV)
Iso5781C (mucoid)
Patient 3
Iso0381 (mucoid)
Iso0668 (mucoid)
Iso0928 (mucoid)
Iso1883 (mucoid)
IsoB2726 (mucoid)
Iso0382 (mucoid)
Iso0929 (other)
Iso1884 (mucoid)
Patient 4
Iso1721 (SCV)
Iso2066 (mucoid)
Iso2419 (other)
IsoB2616 (other)
Iso1722 (other)
IsoB2617 (other)
IsoB2618 (other)
Patient 5
Iso0703 (mucoid)
Iso1076 (SCV)
Iso2100 (other)
Iso5759 (mucoid)
Iso1093 (other)
Patient 6
Iso0925 (SCV)
Iso1858 (other)
Iso2472 (other)
Iso0926 (other)
Iso1859 (mucoid)
Iso2474 (SCV)
Iso0927 (other)
Patient 7
Iso0147 (mucoid)
Iso0440 (SCV)
Iso1149 (other)
Iso1495 (other)
Iso2220 (SCV)**
Iso2479 (SCV)
Iso2744 (mucoid)
Iso0149 (SCV, mucoid)
Iso2221 (other)**
Iso2482 (SCV)
Iso2748 (SCV)
Iso2483 (other)*
Iso2749 (SCV)
* nose smear test
** Q8 (earlier time point)
Patient 8
Iso0004 (other)
Iso0721 (other)
Iso1525 (other)
Iso1879 (mucoid)
Iso2336 (other)
Iso2610 (SCV)
IsoB2733 (other)
Iso2337 (other)
Iso2611 (other)
Patient 9
Iso0070 (mucoid)
Iso0459 (mucoid)
Iso0780 (mucoid)
Iso1435 (mucoid)
Iso1769 (mucoid)
Iso2125 (mucoid)
Iso2411 (mucoid)
Iso2822 (SCV)
IsoB2674 (mucoid)
Iso0071 (mucoid)
Iso2412 (mucoid)
Patient 10
Iso0212 (other)
Iso0696 (SCV)
Iso1512 (other)
Iso2249 (SCV)
Iso2573 (other)
Iso5510-4 (SCV)
Iso0213 (SCV)
Iso1513 (SCV)
Iso2256 (other)
Iso2574 (SCV)
Iso5510-5 (SCV)
Patient 11
Iso1380 (mucoid)
Iso1692 (mucoid)
Iso2059 (mucoid)
Iso2332 (mucoid)
Iso5431-3 (mucoid)
Patient 12
Iso0426 (other)
Iso0731 (other)
Iso1037 (other)
Iso1242 (mucoid)
Iso1664 (mucoid)
Iso2001 (mucoid)
Iso2344 (SCV)
Iso2684 (SCV)
Iso5724 (SCV, mucoid)
Iso0427 (other)
Iso1244 (other)
Iso1665 (mucoid)
Iso2345 (other)
Iso2685 (SCV)
Iso2688 (SCV)
Patient 13
Iso0052 (SCV)
Iso1370 (SCV)
Iso1658 (SCV)
Iso2009 (other)
Iso2545 (SCV)
Iso5708 (mucoid)
Iso1371 (SCV)
Iso2010 (SCV)
Iso2548 (other)
Patient 14
Iso0373 (other)
Iso0711 (mucoid)
Iso1077 (other)
Iso1570 (other)
Iso2420 (mucoid)
Iso5864-1 (SCV)
Iso1078 (other)
Iso1572 (mucoid)
Iso2441 (other)
Iso5864-2 (mucoid)
Iso1079 (other)
Patient 15
Isolate (morphotype)
MIC
AZT
COL
TOB
BAS
AZT
COL
TOB
Iso1397 (mucoid)
Iso1822 (other)
Iso1985 (other)
Iso2322 (mucoid)
Iso2613 (other)
IsoB2734 (SCV)
Iso1823 (other)
Iso1988 (mucoid)
Iso2323 (mucoid)
